# Correction: PCBP1 and PCBP2 both bind heavily oxidized RNA but cause opposing outcomes, suppressing or increasing apoptosis under oxidative conditions

**DOI:** 10.1016/j.jbc.2021.101447

**Published:** 2021-11-26

**Authors:** Takashi Ishii, Tatsuhiro Igawa, Hiroshi Hayakawa, Tsugumi Fujita, Mutsuo Sekiguchi, Yusaku Nakabeppu

The following legend has been corrected. In the legend corresponding to Figure 8B, where it reads:

*B*, cleavage of PARP-1 after H2O2 exposure. Cells (WT, PCBP2-KO, PCBP2-KO + WT and PCBP2-KO + KH1DD) were incubated with 0.2 mM H2O2. At the indicated times, the cells were recovered and boiled in SDS lysis buffer. The samples were subjected to SDS-PAGE followed by Western blotting using an anti-PARP-1 antibody (*top*). The band intensities shown in the blots were measured by Image Quant TL, and the percentage of cleaved PARP1 was determined (*bottom*). Data from three independent experiments are shown as the mean ± SE. A two-way ANOVA was performed as a statistical analysis. ∗∗∗*p* < 0.0001 *versus* all others, ###*p* < 0.0001 *versus* WT.

It should read:

*B*, cleavage of PARP-1 after H2O2 exposure. Cells (WT, PCBP2-KO, PCBP2-KO + WT and PCBP2-KO + KH1DD) were incubated with 0.2 mM H2O2. At the indicated times, the cells were recovered and boiled in SDS lysis buffer. The samples were subjected to SDS-PAGE followed by Western blotting using an anti-PARP-1 antibody (*top*). The band intensities shown in the blots were measured by Image Quant TL, and the percentage of cleaved PARP1 was determined (*bottom*). Data from three independent experiments are shown as the mean ± SE. A two-way ANOVA was performed as a statistical analysis. ∗∗∗*p* < 0.0001 *versus* PCBP2-KO + WT, ∗∗*p* < 0.001 *versus* PCBP2-KO + WT, ###*p* < 0.0001 *versus* WT.

